# Decreased DNA methylation of a CpG site in the *HBAP1* gene in plasma DNA from pregnant women

**DOI:** 10.1371/journal.pone.0198165

**Published:** 2018-05-24

**Authors:** Tanapat Pangeson, Torpong Sanguansermsri, Khwanruedee Mahingsa, Phanchana Sanguansermsri

**Affiliations:** 1 Department of Biochemistry, Faculty of Medical Science, Naresuan University, Phitsanulok, Thailand; 2 Department of Biochemistry, School of Medical Sciences, University of Phayao, Phayao, Thailand; 3 Thalassemia Research Unit, Institute of Human Genetics, University of Phayao, Phayao, Thailand; Centro Cardiologico Monzino, ITALY

## Abstract

**Objective:**

The objective of this study is to identify potential CpG site(s) or DNA methylation pattern(s) in the pseudo α-globin 1 gene (*HBAP1* gene), the gene which locates in α-thalassemia-1 deletion mutation, to differentiate plasma DNA between pregnant and non-pregnant women.

**Method:**

DNA methylation profiles of placenta and peripheral blood from the MethBase database were compared to screen differentially methylated regions. This region was confirmed the differential by methylation-sensitive high resolution melt (MS-HRM) analysis. The differential region was used to compare DNA methylation profile of plasma DNA between pregnant and non-pregnant women by bisulfite amplicon sequencing in three levels: overall, individual CpG sites and individual molecules (DNA methylation patterns).

**Result:**

Using MethBase data, four consecutive CpG sites in the *HBAP1* gene were identified as regions of differential DNA methylation between placenta and peripheral blood. The confirmation by MS-HRM showed the differential DNA methylation profile between the placenta and plasma from non-pregnant women. The comparison of DNA methylation profiles between the plasma of pregnant and non-pregnant women showed that, in the overall levels of the four CpG sites, DNA methylation of pregnant women was detected at lower levels than non-pregnant women. In the individual CpG site level, only the second CpG site showed differential DNA methylation between the groups. In the DNA methylation pattern level, there was no strongly significant differences in DNA methylation patterns between the pregnant and non-pregnant groups.

**Conclusion:**

Our result demonstrated that, in the plasma from pregnant women, only one of the four CpG sites displays a decrease in DNA methylation compared with non-pregnant women. It indicates that this CpG site might be useful for determining the presence or absence of fetal wild-type α-globin gene cluster allele in maternal plasma.

## Introduction

Fetal plasma DNA detection has been widely used for non-invasive prenatal testing (NIPT), e.g. sex determination [1–3], pre-eclampsia [4], preterm delivery [5], aneuploidy and single gene disorders. However, in the case of recessive disorders, DNA sequence identity between fetal pathogenic allele and maternal allele is an obstacle for NIPT.

Barts hydrops fetalis is one of the recessive disorders. The disorder is common in Southeast Asia. It is a cause of fetal death and pre-eclampsia. It results from loss-of-function mutation in α-globin gene cluster. The mutations which are common in Southeast Asia are Thai deletion and Southeast Asian deletion. Both of them are large deletion mutations. The deleted genes in both mutations are α-globin 1 gene (*HBA1*), α-globin 2 gene (*HBA2*) and pseudo α-globin 1 gene (*HBAP1*).

Currently, several developments of Barts hydrops fetalis NIPT focus on how to discriminate the fetal pathogenic allele from maternal background [6–9]. However, no team has successfully discriminated between the fetal recessive allele and the identical maternal allele, independent of genetic polymorphism. This means that the developed methods are not usable when the paternal polymorphism is unknown, or, in the event that the paternal and the maternal polymorphism are the same.

Epigenetic markers seem to be a possible universal marker for discrimination. They are modifications of DNA or histones, with patterns which vary depending on the tissue or cell type. It might be the potential target, because cell-free fetal DNA originates from placenta tissue [10, 11], while the maternal background originates from white blood cells [12]. DNA methylation is one of the epigenetic markers which has shown potential for discriminating fetal cell-free DNA from the maternal background [13, 14]. Differential DNA methylation regions in the α-globin gene cluster might be potential target for Barts hydrops NIPT.

Our preliminary study was performed by comparing DNA methylation profile in the α-globin gene cluster between placenta and peripheral blood. The data from MethBase database (http://smithlabresearch.org/) indicated that the both samples had differential profiles in four CpG sites in the *HBAP1* gene. The profile of placenta was lower than blood. We hypothesized that, while the maternal background was contaminated with fetal plasma DNA, the DNA methylation profiles in the four CpG sites must have decreased compared to the non-pregnant situation.

This study aims to compare DNA methylation profiles in the four CpG sites between plasma DNA from pregnant and non-pregnant women for proving our hypothesis. In addition, it will determine the highly differential DNA methylation pattern(s) or the CpG site(s). The differential pattern(s) or the site(s) will be the potential target for tracing the contamination of fetal normal allele in maternal plasma.

## Materials and methods

### Sample collection

This study was approved by the Naresuan University Institutional Review Board (IRB No.355/58). Informed consent was obtained from all participants. All the consent was written. The participants were non-pregnant women, and women with singleton pregnancies, between 8 and 19 weeks of gestation. Ten milliliters of whole blood was collected in EDTA tubes and the plasma was isolated within 2 hours. The plasma isolation was performed by centrifugation at 3200 rpm for 20 minutes at 4°C. The isolated plasma was then centrifuged at 14000 rpm for 5 minutes at 4°C. The supernatant was re-centrifuged at 14000 rpm for 5 minutes at 4°C.

### DNA extraction and bisulfite conversion

DNA was extracted from 25 mg chorionic villus samples using the QIAamp DNA mini Kit (QIAGEN, Germany) according to the manufacturer’s instructions, with an elution volume of 250 μl. Plasma DNA samples were extracted from 3 ml of the processed plasma using the QIAamp Circulating Nucleic Acid Kit (QIAGEN, Germany) according to the manufacturer’s instructions, with an elution volume of 100 μl. Twenty milliliters of extracted plasma DNA was bisulfite-converted using the EZ DNA Methylation-Gold kit (Zymo Research, USA) according to the manufacturer’s instructions, with an elution volume of 20 μl.

### Methylation-sensitive high resolution melt (MS-HRM)

PCR reactions were performed in a final reaction volume of 25 μL, containing 200 nmol/L of forward primer (5’-TTAAGAAATAATGTAAGTAGGTGGT-3’) and reverse primer (5’-TATTACCTAAATCCACCCACAACTC-3’), 200 μmol/L of each deoxynucleotide triphosphate, 2 μmol/L of SYTO9, 1.5 mmol/L of MgCl_2_, 1 unit of Platinum Taq DNA Polymerase in its supplied buffer (1×), and 3 μl of bisulfite-modified DNA. The thermal cycling conditions on the CFX96 Touch real-time PCR were: 1 cycle at 94°C for 2 minutes, followed by 40 cycles at 94°C for 15 seconds, 57°C for 10 seconds, and 72°C for 10 seconds. After thermal cycling, PCR products were analyzed by melting analysis under the following conditions: 95°C for 30 seconds and 50°C for 1 minute, increasing by 0.5°C every 10 seconds. The data were analyzed in high resolution by the Bio-Rad precision melt analysis software.

### Bisulfite amplicon sequencing

The amplicons from the MS-HRM step were purified with the QIAquick PCR Purification Kit (QIAGEN, Germany). The purified amplicons were used to prepare the library with an Ion Plus Fragment Library Kit (Life Technologies, USA) according to the manufacturer’s instructions, and then amplicon sequencing was performed on an Ion PI Chip and sequenced with the Ion Proton System.

### DNA methylation analysis

In the quality control step, the raw data in FASTQ format were filtered the read length <120bp and quality score >20 using Galaxy tools (https://usegalaxy.org/). The filtered FASTQ data were converted to FASTA format, and then imported to the BiQ Analyzer HT software [15]. The data were aligned against the 129 bp bisulfite reference sequence for mapping DNA methylation patterns in individual reads, and calculating the total DNA methylation level in the individual sample and the DNA methylation level for individual CpG sites. The minimal conversion rate for quality control was set at 1.0.

## Results

### Differential DNA methylation profiles of 4 CpG sites in the *HBAP1* gene between the placenta of pregnant women and the plasma of non-pregnant women

**Fig 1:**
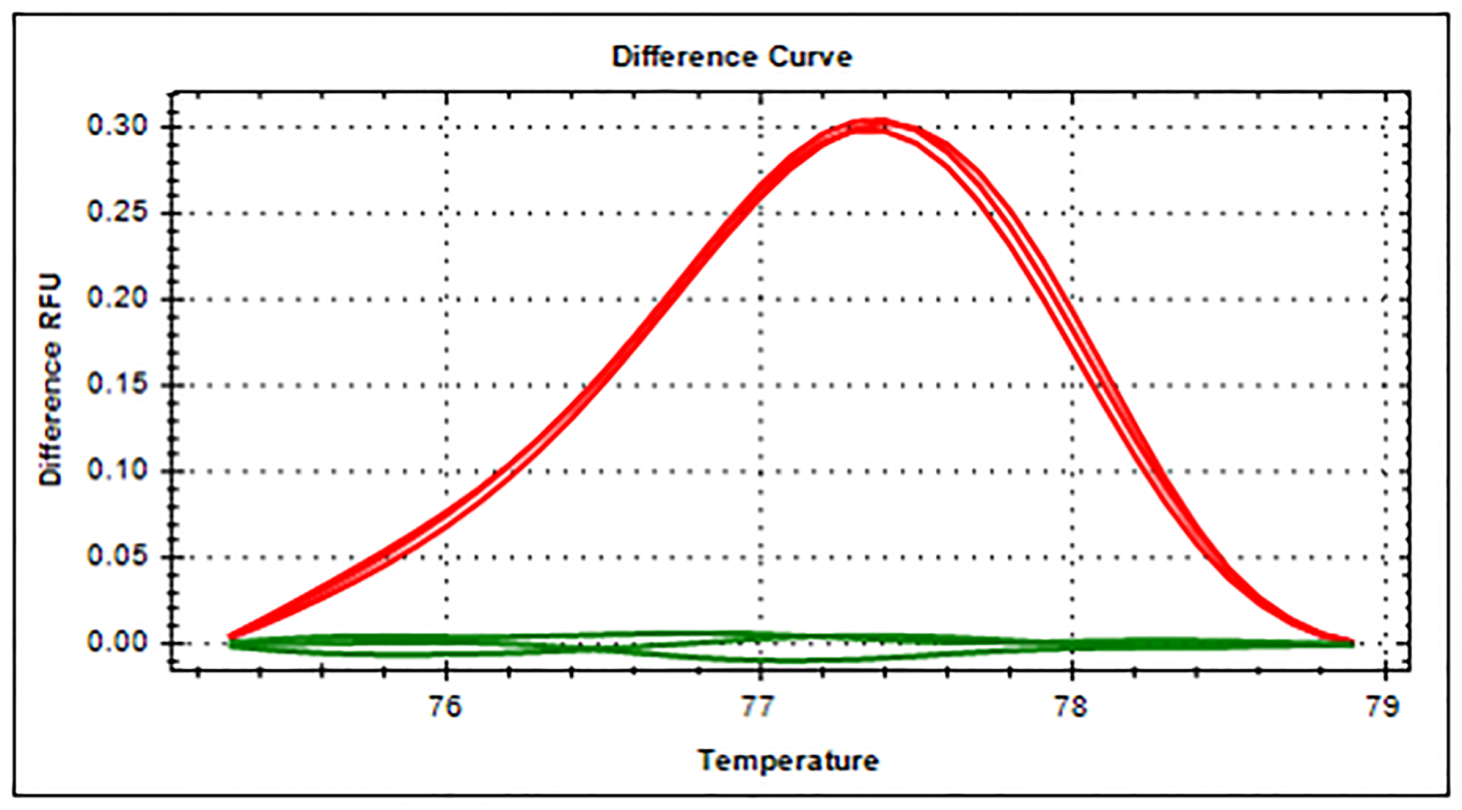
Methylation-sensitive high resolution melting profiles of the *HBAP1* gene between the placenta and the plasma of non-pregnant women are different. Green and red lines represent the placenta and non-pregnant plasma, respectively.

To confirm that regions in the *HBAP1* gene show differential methylation profiles between placenta and plasma DNA, according to the MethBase database, two types of bisulfite amplicon were compared by MS-HRM. A differential methylation profile was found (Fig 1). From previous study, when the DNA sample has higher relative fluorescence units (RFUs) than others, it means that it has a higher DNA methylation level [16]. This implies that the DNA methylation level of the plasma is higher than that of the placenta.

### The differential DNA methylation profile of 4 CpG sites in the *HBAP1* gene between the plasma of pregnant women and that of non-pregnant women

**Fig 2:**
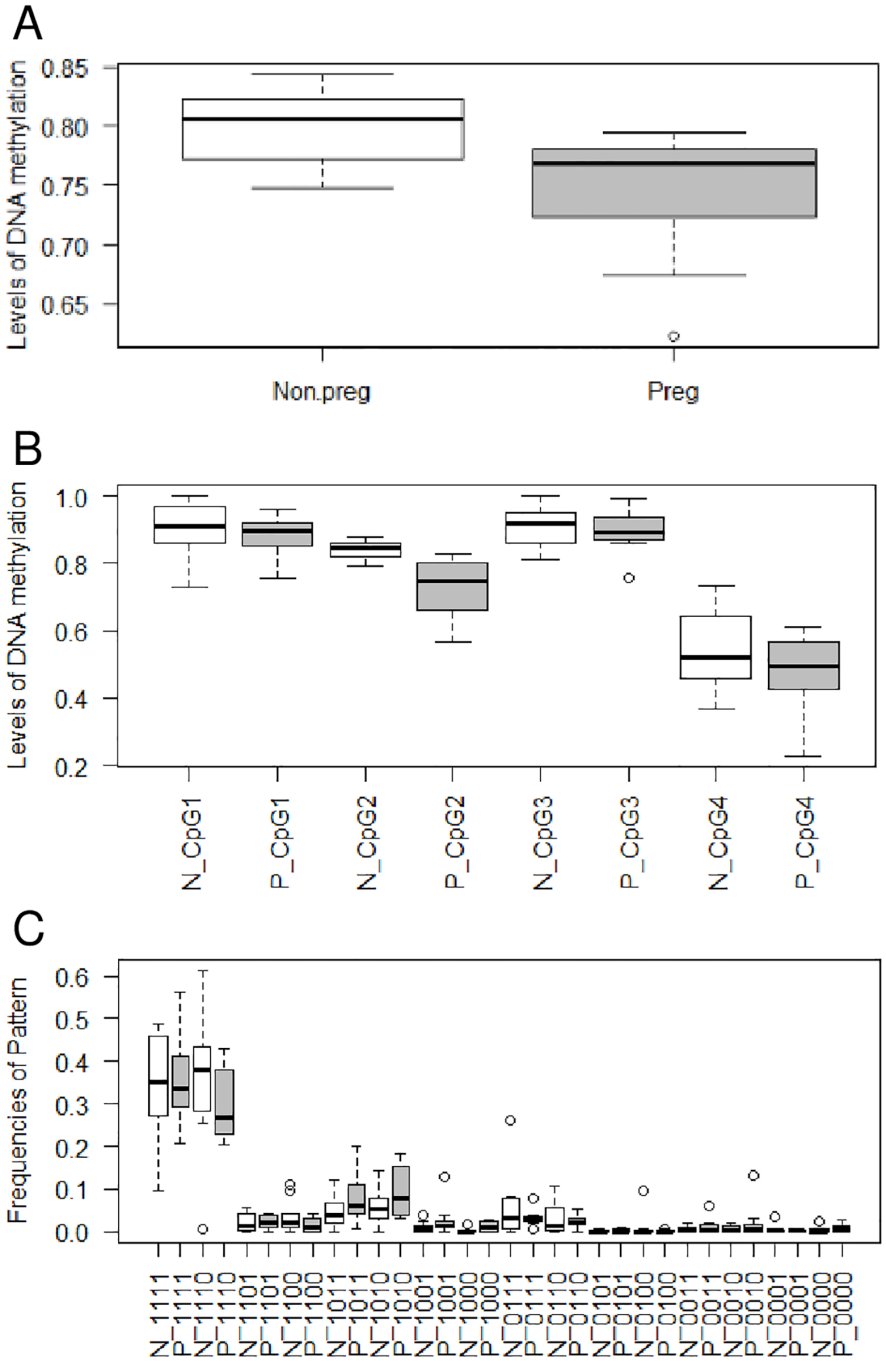
DNA methylation profiles of plasma DNA between pregnant and non-pregnant women were compared at three levels. There are (A) overall, (B) individual CpG sites, and (C) DNA methylation patterns. Grey and white boxes in the boxplot represent the pregnant and non-pregnant groups, respectively. P and N represents the group of pregnant and non-pregnant women, respectively. 0 and 1 represent unmethylated and methylated status for CpG sites, respectively.

More than 280 exported reads for each sample were used to calculate the DNA methylation profile. The mean total DNA methylation level in pregnant and non-pregnant women was 0.745±0.053 and 0.797±0.032, respectively. The two groups were statically different in the t-test (P = 0.023; Fig 2A). Overall, four CpG sites show the ability to differentiate between two groups, as pregnant women show lower DNA methylation levels than non-pregnant women.

The 4 CpG sites were compared between groups. The mean DNA methylation levels of all individual CpG sites in pregnant women were lower than those of non-pregnant women. However, only the pair at the second CpG site was significantly different (P = 0.002). The mean methylation level of the CpG site was 0.723±0.082 and 0.840±0.027 in pregnant and non-pregnant women, respectively (Fig 2B).

The DNA methylation pattern comparison, which was found for each of the two groups, shows that they are not significantly different in the frequencies of patterns. The most significantly different pattern was pattern m1000 (P = 0.097). This pattern shows a higher frequency in non-pregnant women compared to pregnant women. The mean frequency of the pattern in pregnant women and non-pregnant women was 0.012±0.011 and 0.004±0.007, respectively (Fig 2C).

## Discussion

From evidence that cell-free fetal DNA originates from placenta, we hypothesized that when the maternal plasma is contaminated with fetal DNA, the DNA methylation level in the locus is changed from a non-pregnant situation. In this study, we focus on the α-globin gene cluster locus, which are deleted in α-thalassemia-1 deletions. The mutation is a cause of Barts hydrops fetalis. Therefore, if the fetal α-globin gene cluster allele is detected in maternal plasma, it means that the fetus is not Barts hydrops fetalis.

In this study, the marker for detecting fetal wild-type allele were found using the following method: In brief, we located the region of interest, which was confirmed as potential and then used to compare DNA methylation between plasma DNA with or without contaminating fetal DNA. Non-pregnant plasma was used to represent maternal plasma without contaminating fetal DNA and pregnant plasma represented the contaminated maternal plasma.

The regions of interest were screened using the regions which overlap between four major α-thalassemia-1 deletions (Southeast Asian, Thai, Filipino and Mediterranean mutation)[17]. The DNA methylation profiles of placenta and blood from the database were used for screening the highly differential DNA methylation region for finding the potential target. In the database, four CpG sites of interest in the *HBAP1* gene show differential DNA methylation levels in both tissues (S1 Fig). These sites are relatively hypo-methylated in the placenta and hyper-methylated in the blood.

Similarly to the use of the database, when the MS-HRM was used for confirmation, the result showed differential DNA methylation, implying that placenta has relatively lower methylation levels than blood. The differential emphasized the potential of this region for use as a target for discriminating between pregnant and non-pregnant plasma.

According to relative hypo-methylation in placenta, the result from comparing pregnant and non-pregnant plasma showed that the overall DNA methylation level in the plasma DNA of pregnant women was lower than the other group. The lower DNA methylation level in pregnant women might result from the contamination of hypo-methylated placental DNA.

As mentioned previously, in the database, the four CpG sites of placenta showed lower methylation than blood. When maternal plasma was contaminated with placental DNA, all four CpG sites should display lower DNA methylation than non-pregnant plasma. However, our result contradicts the database, as only the second CpG site of pregnant plasma had a clearly decreased DNA methylation level relative to the non-pregnant group.

The cause of the specific decrease of DNA methylation at the second CpG site might be from DNA fragmentation. Before dispersal of the placental DNA in maternal circulation, the DNA was fragmented by physical separation and endonuclease. If the process involves a preferential breakpoint of fragmentation, the consequence is a bias of primer attachment and a bias in the PCR step. The second CpG site without the methyl group might have higher stability for the PCR process than the site with the methyl group.

Otherwise, based on the study by Skvortsova, which suggested that methylated DNA circulates in the blood for longer than unmethylated DNA [18], the unmethylated second CpG sites might be less affected by clearance than the other unmethylated sites. This might result in a higher population of the unmethylated second CpG DNA. This phenomenon is contradicted by the study of Puszyk [19], which suggests that the methylation status of a DNA sequence has no effect on its steady-state concentration in the cell-free DNA component of plasma. Nevertheless, their study focused on the non-pregnant group, while fetal and maternal DNA in our study might have distinct clearance.

Finally, the DNA methylation patterns in both groups were extracted. The results indicate that, in this region, no placenta-specific DNA methylation patterns were found. Thus, the DNA methylation pattern of this region cannot be applied to discriminate fetal DNA from the maternal background.

In conclusion, our results suggest that the decreasing DNA methylation level at the second CpG site of this region can distinguish plasma DNA of pregnant women from that of non-pregnant women. In our future work, we could use this target to rule out those pregnant women who are carrying a fetus with the wild-type allele of the α-globin gene cluster (αα/αα or αα/—) from null allele pregnancies (—/—). We hypothesize that the plasma of null allele pregnancies might not be decreased, but rather, their DNA methylation might be the same level as non-pregnant women.

## Supporting information

S1 Fig DNA methylation level of the 4 CpG sites in MethBase database. (DOCX)

S2 Fig DNA methylation profile of individual samples. (DOCX)

S3 Fig T-test analysis for comparing paired CpG sites between plasma DNA of pregnant and non-pregnant women. (DOCX)
