# Supplementary figures and images for: Decreased DNA methylation of a CpG site in the HBAP1 gene in plasma DNA from pregnant women
Source: PLoS One. 2018 May 24;13(5):e0198165. doi: 10.1371/journal.pone.0198165 (PMC5967787; doi:10.1371/journal.pone.0198165)

Supplementary1


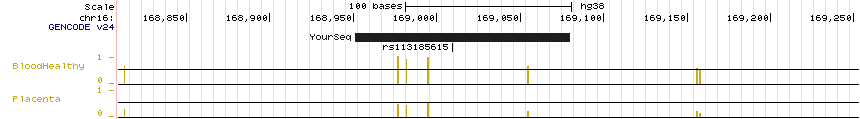

Supplement: S1 Fig — (DOCX) [file pone.0198165.s001.docx]
