# Supplementary material for: Decreased DNA methylation of a CpG site in the HBAP1 gene in plasma DNA from pregnant women
Source: PLoS One. 2018 May 24;13(5):e0198165. doi: 10.1371/journal.pone.0198165 (PMC5967787; doi:10.1371/journal.pone.0198165)

| N01  Amplicon | Sequence |
| --- | --- |
| Sample | N01 |
| Loaded reads | 11335 |
| Filtered out reads | 2925 |
| Exported reads | 8410 |
| Mean methylation level | 0.823 |
| Standard deviation | 0.184 |
| Mean conversion rate | 1.000 |
| Elapsed time | 0 min 24 sec |


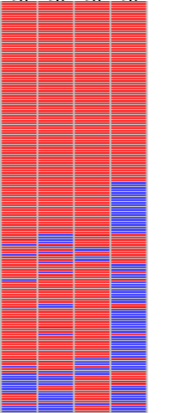

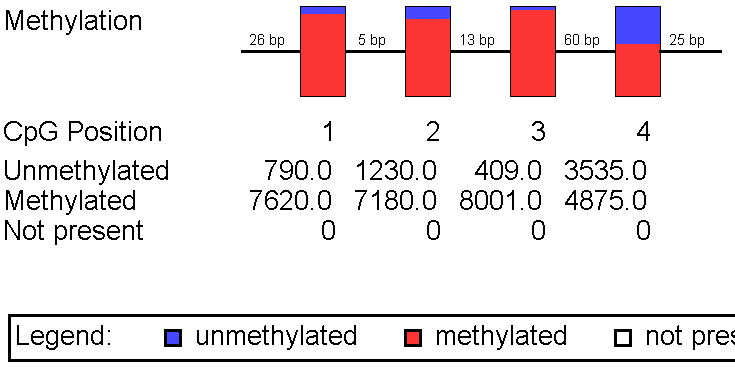


| N02  Amplicon | Sequence |
| --- | --- |
| Sample | N02 |
| Loaded reads | 807 |
| Filtered out reads | 318 |
| Exported reads | 489 |
| Mean methylation level | 0.752 |
| Standard deviation | 0.211 |
| Mean conversion rate | 1.000 |
| Elapsed time | 0 min 1 sec |


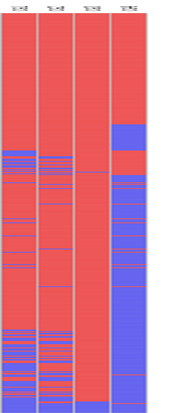

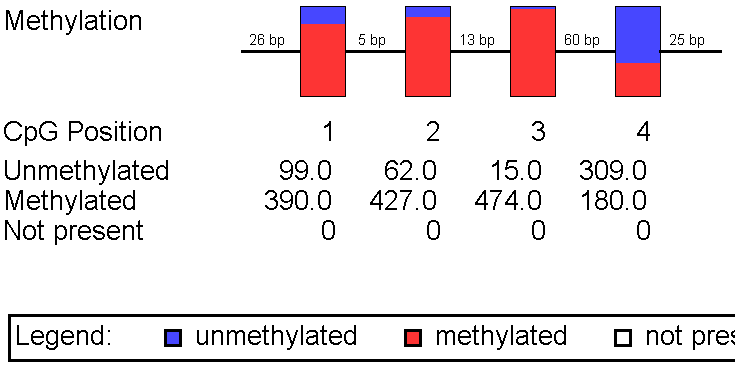


| N03  Amplicon | Sequence |
| --- | --- |
| Sample | N03 |
| Loaded reads | 2085 |
| Filtered out reads | 872 |
| Exported reads | 1213 |
| Mean methylation level | 0.820 |
| Standard deviation | 0.163 |
| Mean conversion rate | 1.000 |
| Elapsed time | 0 min 4 sec |


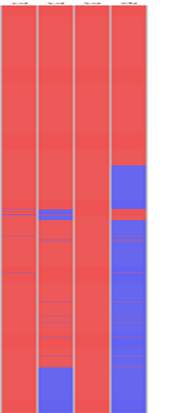

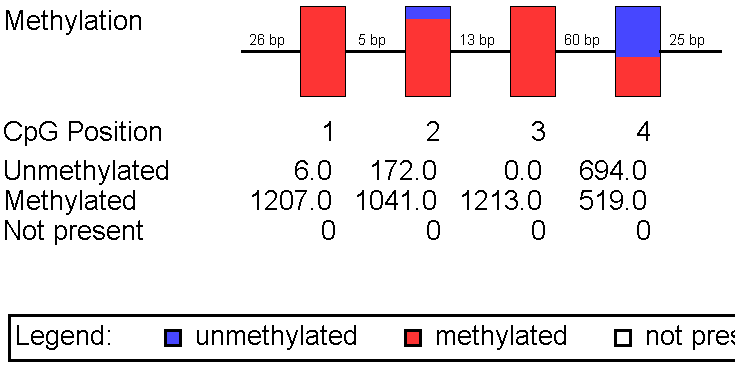


| N04  Amplicon | Sequence |
| --- | --- |
| Sample | N04 |
| Loaded reads | 11852 |
| Filtered out reads | 2731 |
| Exported reads | 9121 |
| Mean methylation level | 0.748 |
| Standard deviation | 0.218 |
| Mean conversion rate | 1.000 |
| Elapsed time | 0 min 27 sec |


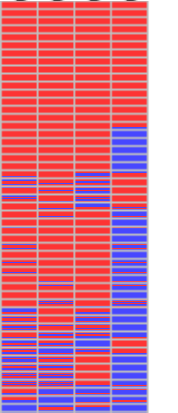

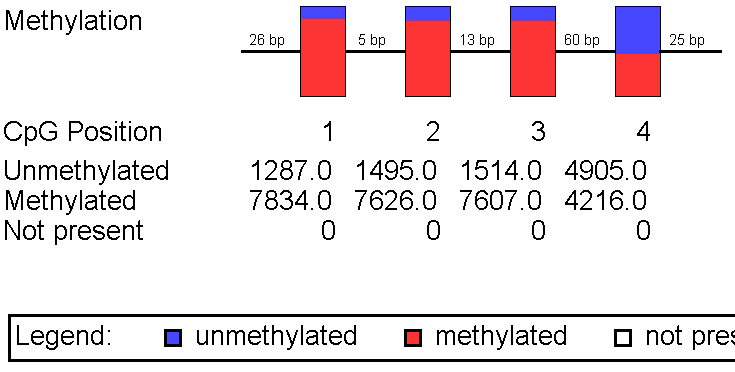


| N05  Amplicon | Sequence |
| --- | --- |
| Sample | N05 |
| Loaded reads | 8228 |
| Filtered out reads | 1961 |
| Exported reads | 6267 |
| Mean methylation level | 0.843 |
| Standard deviation | 0.176 |
| Mean conversion rate | 1.000 |
| Elapsed time | 0 min 18 sec |


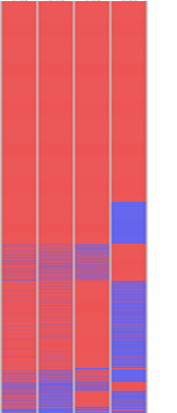

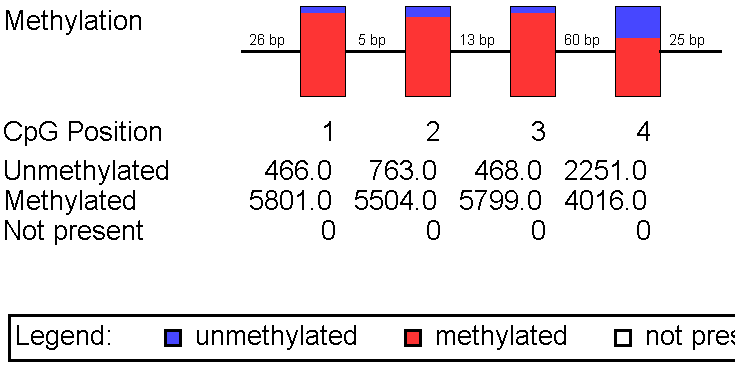


| N06  Amplicon | Sequence |
| --- | --- |
| Sample | N06 |
| Loaded reads | 850 |
| Filtered out reads | 265 |
| Exported reads | 585 |
| Mean methylation level | 0.812 |
| Standard deviation | 0.222 |
| Mean conversion rate | 1.000 |
| Elapsed time | 0 min 1 sec |


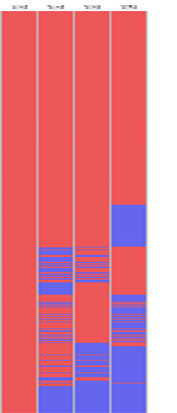

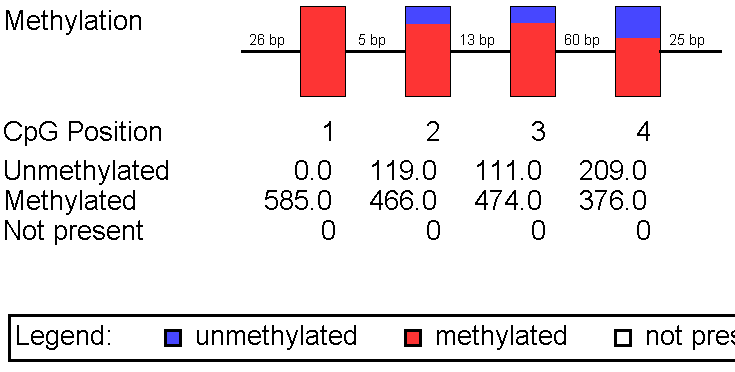


| N07  Amplicon | Sequence |
| --- | --- |
| Sample | N08 |
| Loaded reads | 1962 |
| Filtered out reads | 1111 |
| Exported reads | 851 |
| Mean methylation level | 0.799 |
| Standard deviation | 0.207 |
| Mean conversion rate | 1.000 |
| Elapsed time | 0 min 4 sec |


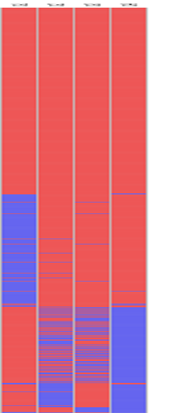

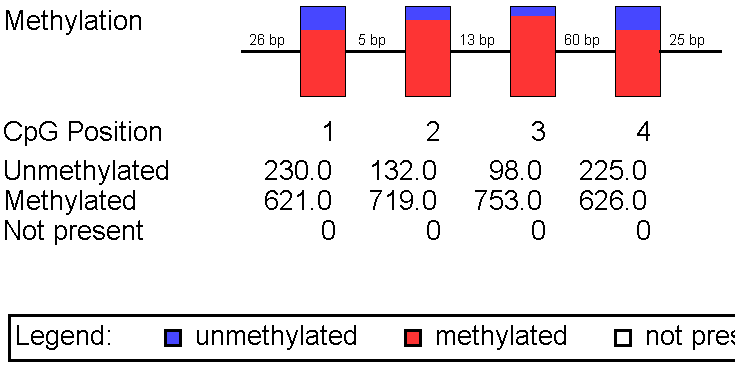


| N08  Amplicon | Sequence |
| --- | --- |
| Sample | N09 |
| Loaded reads | 691 |
| Filtered out reads | 285 |
| Exported reads | 406 |
| Mean methylation level | 0.773 |
| Standard deviation | 0.178 |
| Mean conversion rate | 1.000 |
| Elapsed time | 0 min 1 sec |


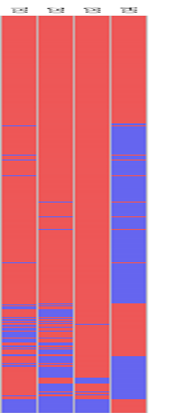

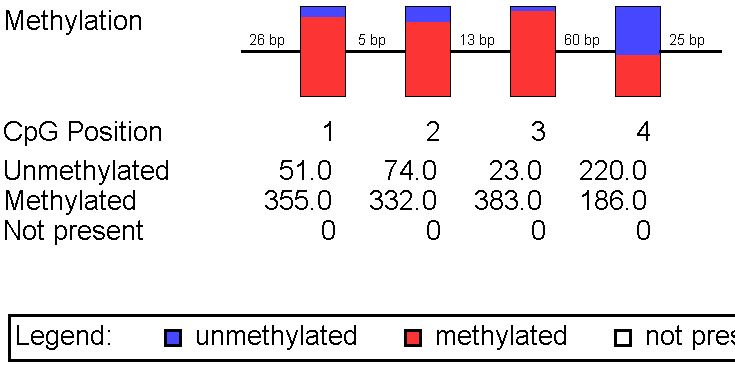


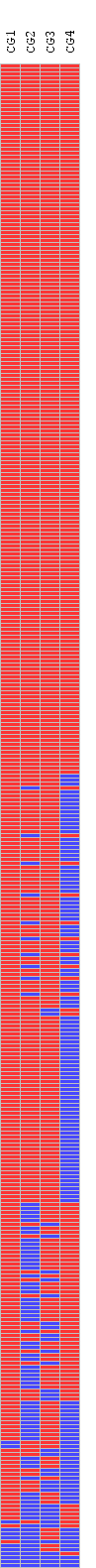
N09

| Amplicon | Sequence |
| --- | --- |
| Sample | N10 |
| Loaded reads | 539 |
| Filtered out reads | 160 |
| Exported reads | 379 |
| Mean methylation level | 0.832 |
| Standard deviation | 0.193 |
| Mean conversion rate | 1.000 |
| Elapsed time | 0 min 1 sec |


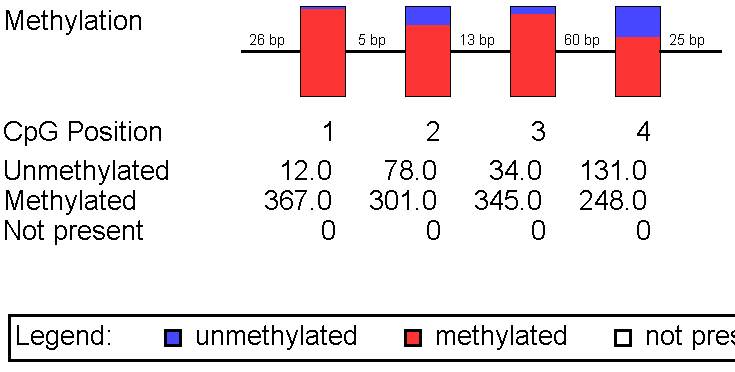


| N10  Amplicon | Sequence |
| --- | --- |
| Sample | N11 |
| Loaded reads | 412 |
| Filtered out reads | 129 |
| Exported reads | 283 |
| Mean methylation level | 0.772 |
| Standard deviation | 0.178 |
| Mean conversion rate | 1.000 |
| Elapsed time | 0 min 0 sec |


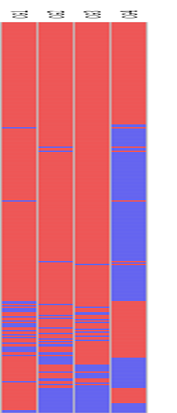

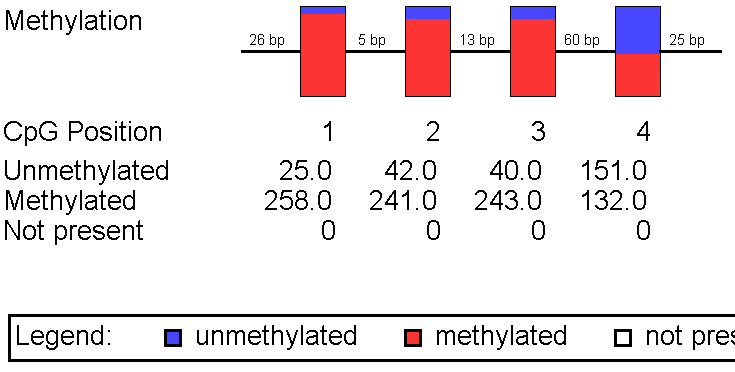


| P01  Amplicon | Sequence |
| --- | --- |
| Sample | P01 |
| Loaded reads | 1030 |
| Filtered out reads | 401 |
| Exported reads | 629 |
| Mean methylation level | 0.760 |
| Standard deviation | 0.159 |
| Mean conversion rate | 1.000 |
| Elapsed time | 0 min 2 sec |


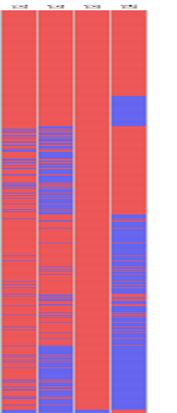

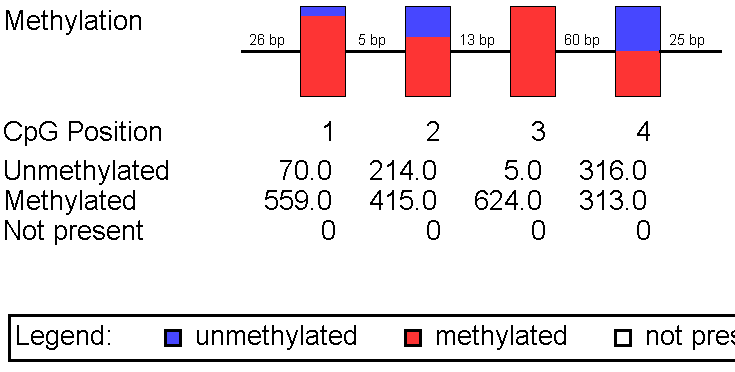


| P02  Amplicon | Sequence |
| --- | --- |
| Sample | P02 |
| Loaded reads | 946 |
| Filtered out reads | 299 |
| Exported reads | 647 |
| Mean methylation level | 0.723 |
| Standard deviation | 0.181 |
| Mean conversion rate | 1.000 |
| Elapsed time | 0 min 2 sec |


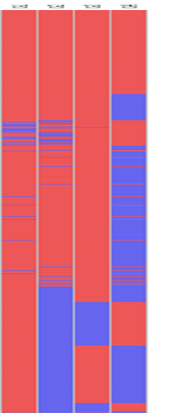

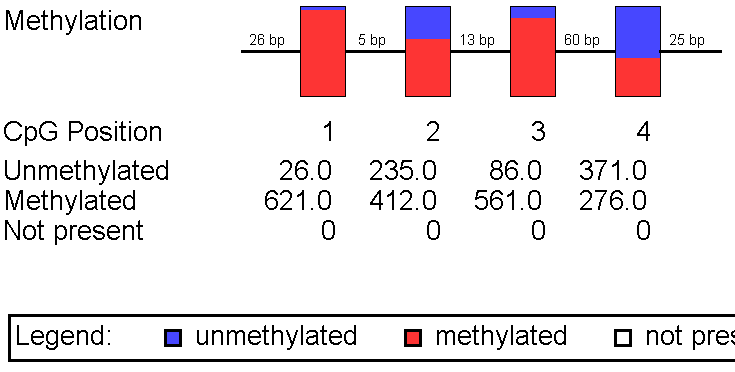


| P03  Amplicon | Sequence |
| --- | --- |
| Sample | P03 |
| Loaded reads | 678 |
| Filtered out reads | 231 |
| Exported reads | 447 |
| Mean methylation level | 0.780 |
| Standard deviation | 0.216 |
| Mean conversion rate | 1.000 |
| Elapsed time | 0 min 1 sec |


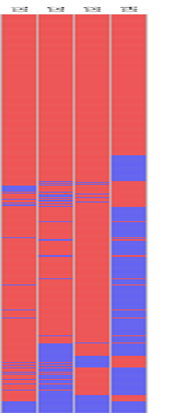

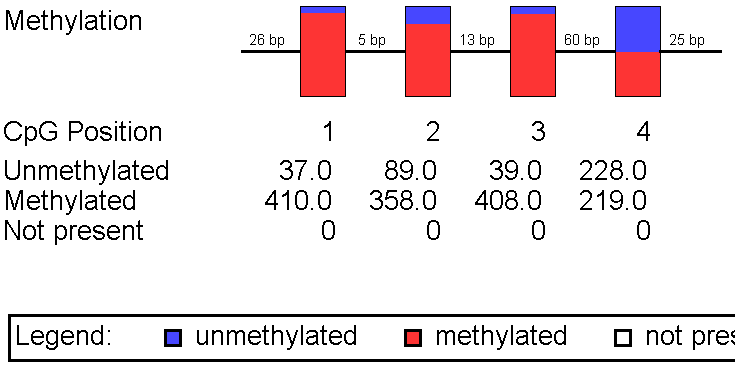


| P04  Amplicon | Sequence |
| --- | --- |
| Sample | P04 |
| Loaded reads | 728 |
| Filtered out reads | 190 |
| Exported reads | 538 |
| Mean methylation level | 0.757 |
| Standard deviation | 0.206 |
| Mean conversion rate | 1.000 |
| Elapsed time | 0 min 1 sec |


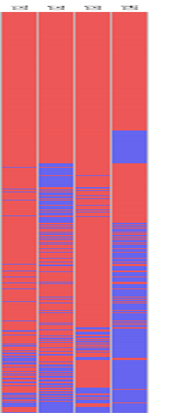

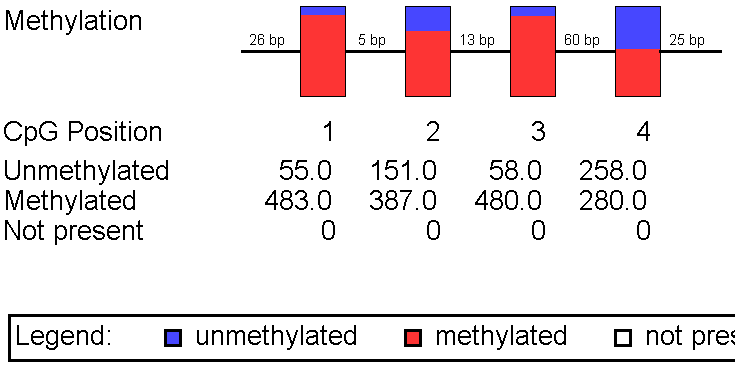


| P05  Amplicon | Sequence |
| --- | --- |
| Sample | P05 |
| Loaded reads | 1291 |
| Filtered out reads | 353 |
| Exported reads | 938 |
| Mean methylation level | 0.777 |
| Standard deviation | 0.249 |
| Mean conversion rate | 1.000 |
| Elapsed time | 0 min 3 sec |


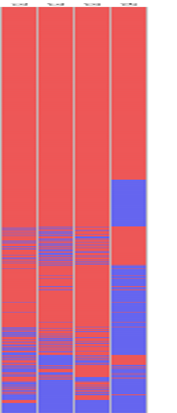

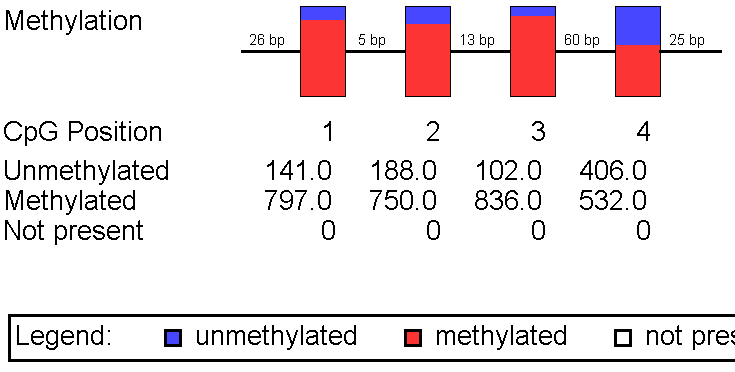


| P06  Amplicon | Sequence |
| --- | --- |
| Sample | P06 |
| Loaded reads | 1823 |
| Filtered out reads | 490 |
| Exported reads | 1333 |
| Mean methylation level | 0.621 |
| Standard deviation | 0.211 |
| Mean conversion rate | 1.000 |
| Elapsed time | 0 min 3 sec |


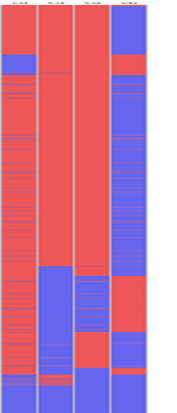

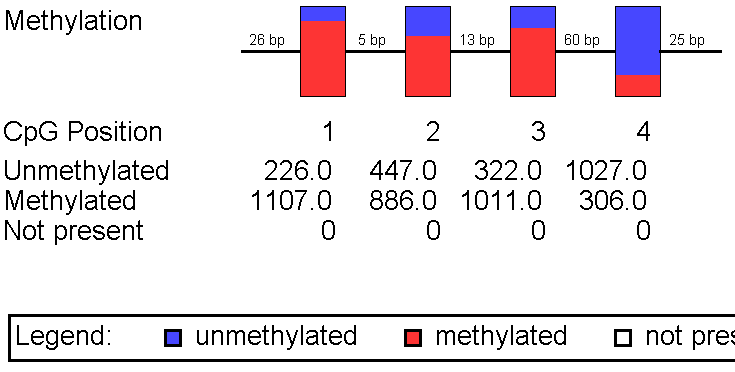


| P07  Amplicon | Sequence |
| --- | --- |
| Sample | P08 |
| Loaded reads | 5408 |
| Filtered out reads | 1189 |
| Exported reads | 4219 |
| Mean methylation level | 0.787 |
| Standard deviation | 0.219 |
| Mean conversion rate | 1.000 |
| Elapsed time | 0 min 11 sec |


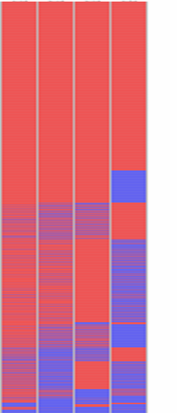

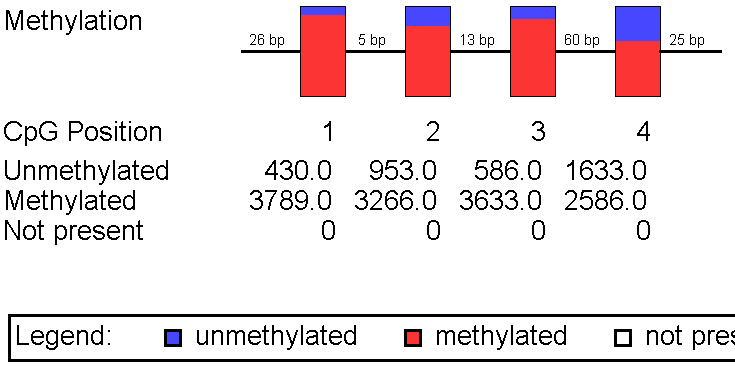


| P08  Amplicon | Sequence |
| --- | --- |
| Sample | P09 |
| Loaded reads | 437 |
| Filtered out reads | 101 |
| Exported reads | 336 |
| Mean methylation level | 0.776 |
| Standard deviation | 0.182 |
| Mean conversion rate | 1.000 |
| Elapsed time | 0 min 0 sec |


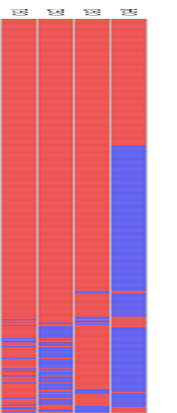

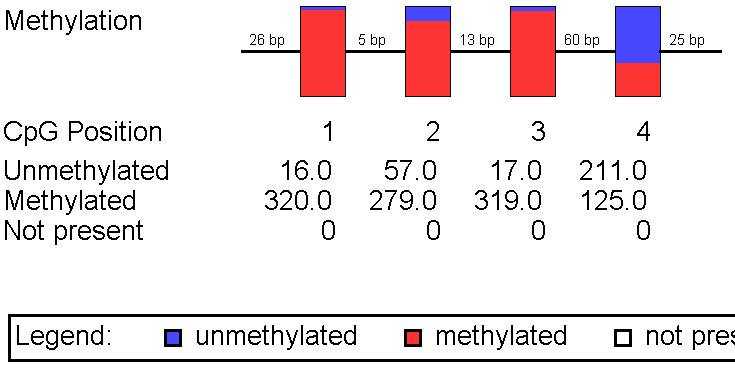


| P09  Amplicon | Sequence |
| --- | --- |
| Sample | P10 |
| Loaded reads | 326 |
| Filtered out reads | 81 |
| Exported reads | 245 |
| Mean methylation level | 0.674 |
| Standard deviation | 0.268 |
| Mean conversion rate | 1.000 |
| Elapsed time | 0 min 0 sec |


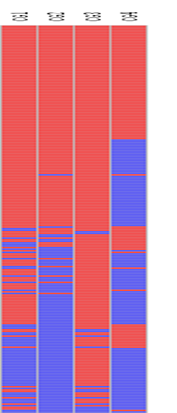

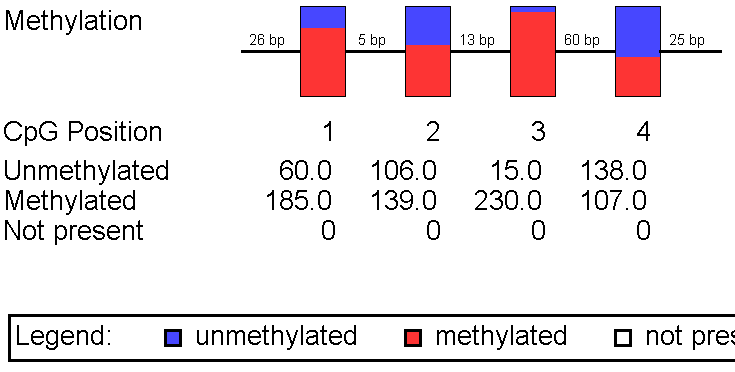


| P10  Amplicon | Sequence |
| --- | --- |
| Sample | P11 |
| Loaded reads | 440 |
| Filtered out reads | 96 |
| Exported reads | 344 |
| Mean methylation level | 0.794 |
| Standard deviation | 0.217 |
| Mean conversion rate | 1.000 |
| Elapsed time | 0 min 0 sec |


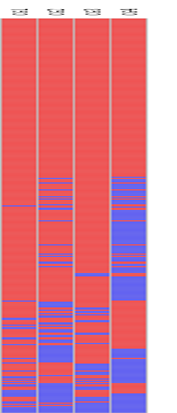

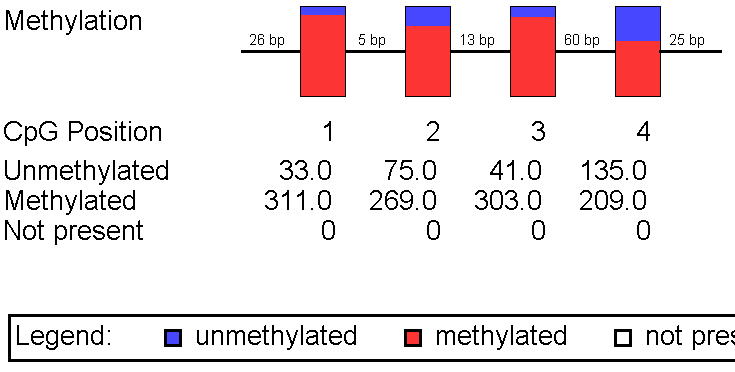

Supplement: S2 Fig — (DOCX) [file pone.0198165.s002.docx]
