# Supplementary material for: Decreased DNA methylation of a CpG site in the HBAP1 gene in plasma DNA from pregnant women
Source: PLoS One. 2018 May 24;13(5):e0198165. doi: 10.1371/journal.pone.0198165 (PMC5967787; doi:10.1371/journal.pone.0198165)
Supplement: S3 Fig — (DOCX) [file pone.0198165.s003.docx]

CpG1

| t-Test: Two-Sample Assuming Unequal Variances | | |
| --- | --- | --- |
|  |  |  |
|  | *Non-preg* | *Preg* |
| Mean | 0.896732 | 0.885329 |
| Variance | 0.007363 | 0.003665 |
| Observations | 10 | 10 |
| Hypothesized Mean Difference | 0 |  |
| df | 16 |  |
| t Stat | 0.343366 |  |
| P(T<=t) one-tail | 0.367897 |  |
| t Critical one-tail | 1.745884 |  |
| P(T<=t) two-tail | 0.735793 |  |
| t Critical two-tail | 2.119905 |  |

CpG2

| t-Test: Two-Sample Assuming Unequal Variances | | |
| --- | --- | --- |
|  |  |  |
|  | *Non-preg* | *Preg* |
| Mean | 0.840449 | 0.723483 |
| Variance | 0.00086 | 0.007624 |
| Observations | 10 | 10 |
| Hypothesized Mean Difference | 0 |  |
| df | 11 |  |
| t Stat | 4.015682 |  |
| P(T<=t) one-tail | 0.001016 |  |
| t Critical one-tail | 1.795885 |  |
| P(T<=t) two-tail | 0.002031 |  |
| t Critical two-tail | 2.200985 |  |

CpG3

| t-Test: Two-Sample Assuming Unequal Variances | | |
| --- | --- | --- |
|  |  |  |
|  | *Non-preg* | *Preg* |
| Mean | 0.908742 | 0.894387 |
| Variance | 0.003739 | 0.003934 |
| Observations | 10 | 10 |
| Hypothesized Mean Difference | 0 |  |
| df | 18 |  |
| t Stat | 0.518209 |  |
| P(T<=t) one-tail | 0.305312 |  |
| t Critical one-tail | 1.734064 |  |
| P(T<=t) two-tail | 0.610624 |  |
| t Critical two-tail | 2.100922 |  |

CpG4

| t-Test: Two-Sample Assuming Unequal Variances | | |
| --- | --- | --- |
|  |  |  |
|  | *Non-preg* | *Preg* |
| Mean | 0.543593 | 0.476056 |
| Variance | 0.014865 | 0.013615 |
| Observations | 10 | 10 |
| Hypothesized Mean Difference | 0 |  |
| df | 18 |  |
| t Stat | 1.265517 |  |
| P(T<=t) one-tail | 0.110911 |  |
| t Critical one-tail | 1.734064 |  |
| P(T<=t) two-tail | 0.221823 |  |
| t Critical two-tail | 2.100922 |  |
